# Granulocyte, granulocyte–macrophage, and macrophage colony-stimulating factors can stimulate the invasive capacity of human lung cancer cells

**DOI:** 10.1038/sj.bjc.6690009

**Published:** 1999-09-24

**Authors:** X-H Pei, Y Nakanishi, K Takayama, F Bai, N Hara

**Affiliations:** Research Institute for Diseases of the Chest, Faculty of Medicine, Kyushu University, Fukuoka, Japan

**Keywords:** colony-stimulating factors, lung cancer, invasion, uPA, MMPs

## Abstract

We and other researchers have previously found that colony-stimulating factors (CSFs), which generally include granulocyte colony-stimulating factor (G-CSF), granulocyte–macrophage colony-stimulating factor (GM-CSF) and macrophage colony-stimulating factor (M-CSF), promote invasion by lung cancer cells. In the present study, we studied the effects of these CSFs on gelatinase production, urokinase plasminogen activator (uPA) production and their activity in human lung cancer cells. Gelatin zymographs of conditioned media derived from human lung adenocarcinoma cell lines revealed two major bands of gelatinase activity at 68 and 92 kDa, which were characterized as matrix metalloproteinase (MMP)-2 and MMP-9 respectively. Treatment with CSFs increased the 68- and 92-kDa activity and converted some of a 92-kDa proenzyme to an 82-kDa enzyme that was consistent with an active form of the MMP-9. Plasminogen activator zymographs of the conditioned media from the cancer cells showed that CSF treatment resulted in an increase in a 48–55 kDa plasminogen-dependent gelatinolytic activity that was characterized as human uPA. The conditioned medium from the cancer cells treated with CSFs stimulated the conversion of plasminogen to plasmin, providing a direct demonstration of the ability of enhanced uPA to increase plasmin-dependent proteolysis. The enhanced invasive behaviour of the cancer cells stimulated by CSFs was well correlated with the increase in MMPs and uPA activities. These data suggest that the enhanced production of extracellular matrix-degrading proteinases by the cancer cells in response to CSF treatment may represent a biochemical mechanism which promotes the invasive behaviour of the cancer cells. © 1999 Cancer Research Campaign


*British Journal of Cancer* (1999) **79**(1), 40–46
© 1999 Cancer Research Campaign

### Granulocyte, granulocyte–macrophage, and macrophage colony-stimulating factors can stimulate the invasive capacity of human lung cancer cells

**X-H Pei, Y Nakanishi, K Takayama, F Bai and N Hara**

Research Institute for Diseases of the Chest, Faculty of Medicine, Kyushu University, Fukuoka, Japan

**Summary** We and other researchers have previously found that colony-stimulating factors (CSFs), which generally include granulocyte colony-stimulating factor (G-CSF), granulocyte–macrophage colony-stimulating factor (GM-CSF) and macrophage colony-stimulating factor (M-CSF), promote invasion by lung cancer cells. In the present study, we studied the effects of these CSFs on gelatinase production, urokinase plasminogen activator (uPA) production and their activity in human lung cancer cells. Gelatin zymographs of conditioned media derived from human lung adenocarcinoma cell lines revealed two major bands of gelatinase activity at 68 and 92 kDa, which were characterized as matrix metalloproteinase (MMP)-2 and MMP-9 respectively. Treatment with CSFs increased the 68- and 92-kDa activity and converted some of a 92-kDa proenzyme to an 82-kDa enzyme that was consistent with an active form of the MMP-9. Plasminogen activator zymographs of the conditioned media from the cancer cells showed that CSF treatment resulted in an increase in a 48–55 kDa plasminogen-dependent gelatinolytic activity that was characterized as human uPA. The conditioned medium from the cancer cells treated with CSFs stimulated the conversion of plasminogen to plasmin, providing a direct demonstration of the ability of enhanced uPA to increase plasmin-dependent proteolysis. The enhanced invasive behaviour of the cancer cells stimulated by CSFs was well correlated with the increase in MMPs and uPA activities. These data suggest that the enhanced production of extracellular matrix-degrading proteinases by the cancer cells in response to CSF treatment may represent a biochemical mechanism which promotes the invasive behaviour of the cancer cells.

**Keywords:** colony-stimulating factors; lung cancer; invasion; uPA; MMPs

The complicated process of metastasis requires tumour cells to successfully traverse a number of barriers including tumour and host vasculature, basement membranes of blood vessels and organ parenchyma, and the interstitial stroma. These steps involve adhesion, local proteolysis, migration and growth (Liotta et al, 1991). Several tumour and normal cell-derived cytokines such as hepatocyte growth factor (Jeffers et al, 1996; Weimar et al, 1997), transforming growth factor (Teti et al, 1997), and epidermal growth factor (Zhang et al, 1997) have been shown to stimulate tumour cell adhesion and proteolysis as well as migration (Herlyn and Malkowicz, 1991; Ries and Petrides, 1995).

Matrix-degrading proteinases are essential to successful tumour cell metastasis. Secretion of matrix metalloproteinases (MMPs) and plasminogen activators (PAs) into the surrounding microenvironment is essential for facilitating tumour cell invasion and metastasis via proteolytic degradation of the extracellular matrix (ECM), and tumour aggressiveness has been positively correlated with the level of secreted proteinases (Stetler-Stevenson, 1990; Testa and Quigley, 1990; Liotta et al, 1991).

Colony-stimulating factors (CSFs), classically considered to function in the regulation of haematopoiesis, are secreted by haematopoietic cells and some non-haematopoietic cells (Young et al, 1993; Harmenberg et al, 1994; Chambers et al, 1995; Pei et al, 1996). Their receptors were also found to be expressed on non-haematopoietic tumour cells. Granulocyte–macrophage colony-stimulating factor (GM-CSF) and macrophage colony-stimulating factor (M-CSF) have been reported to stimulate the metastatic properties of lung carcinoma cell lines (Young et al, 1993; Chambers et al, 1995). Recently, we demonstrated that granulocyte colony-stimulating factor (G-CSF), another colony-stimulating factor, also promoted invasion by human lung cancer cell lines in vitro (Pei et al, 1996). However, the role of these CSFs in regulating proteinase production and activity in human lung cancer cells has not been well documented.

ECM degradation must involve the action of an array of hydrolytic enzymes such as serine proteinases, metalloproteinases and cysteine proteinases. The expression of these enzymes is regulated by several agents, including tumour promoters, oncogenes, growth factors and cytokines (Stetler-Stevenson, 1990; Testa and Quigley, 1990; Mignatti and Rifkin, 1993). These enzymes appear to act in concert via a cascade of proteolytic events whose end result is the generation of a broad spectrum of proteolytic activities. The PA–plasmin system appears to play a pivotal role in this cascade. An important feature of the PA–plasmin modulatory system is the amplification achieved by the conversion of plasminogen to plasmin. The concentration of circulating plasminogen is relatively high (~200 $\mu g\ ml^{-1}$), and in humans ~40% of the plasminogen is located in extravascular sites (Mignatti and Rifkin, 1993). Thus, the production of small amounts of PA can result in high local concentrations of plasmin. PAs are secreted as single-chain proenzymes [pro-urokinase plasminogen activator or pro-tissue plasminogen activator (pro-uPA or pro-tPA)] that are

*Received 10 December 1997*
*Revised 5 May 1998*
*Accepted 12 May 1998*

*Correspondence to:* Y Nakanishi, Research Institute for Diseases of the Chest, Faculty of Medicine, Kyushu University, 3-1-1 Maidashi, Higashiku, Fukuoka 812–82, Japan converted to the two-chain form by limit proteolysis. Trace amounts of plasmin are able to activate pro-uPA (Petersen et al, 1988), thus generating a self-maintained feedback mechanism of pro-uPA and plasminogen activation. Plasmin degrades several ECM components and at the same time activates the procollagenases and prostromelysins. Thus, production of even small amounts of PA results in the generation of high local concentration of broad-spectrum enzymes.

In the present study, we showed that CSFs promoted invasion by human lung cancer cells. We characterized the PA and MMP activities of the cancer cells and found that treatment with CSFs enhanced production of specific PA and MMPs by these cells. The enhanced proteolytic capability of the tumour cells suggests a biochemical mechanism by which increased invasion stimulated by CSFs may be mediated.

### MATERIALS AND METHODS

### Cells and chemicals

Human lung adenocarcinoma cell lines, PC-9 and A549, were maintained in RPMI-1640 medium containing 5% fetal bovine serum (FBS, CC Laboratories, Cleveland, OH, USA). A human lung fibroblast cell line, MRC-5, was grown in basal medium Eagle (BME) medium supplemented with 10% FBS. Before and during the experiments, cell viability was determined by trypan blue exclusion. Only cell suspensions exhibiting > 95% viability were used. Nartograstim, human recombinant granulocyte colony-stimulating factor, was obtained from Kyowa Hakko Kogyo (Tokyo, Japan). The other agents kindly provided were; rhGM-CSF (Kirin Brewery, Tokyo, Japan), M-CSF (Morinaga Milk Industry, Tokyo, Japan). Gelatin was purchased from Sigma (MO, USA). Plasminogen was from Gelco Diagnostics (Shreveport, LA, USA). Monoclonal antibodies to MMPs, corresponding to the amino acid sequences of the carboxy-terminal domains (residues 524–539, VTPRDKPMGPLLVATF for MMP-2; and residues 624–644, RSAEVDRMFPGVPLDTHD for MMP-9), were from Fuji Chemical Industries (Fukuyama, Japan). Polyclonal antibodies directed against uPA and tPA from TechnoClone (Vienna, Austria). Human uPA standard from JCR Pharmaceuticals (Kobe, Japan). Hybond ECL nitrocellulose membrane and ECL photodetection kit were purchased from Amersham Life Science (Buckinghamshire, UK). NuSerum (10%) were from Becton Dickinson (Bedford, MA, USA). The Biocoat Matrigel Invasion Chamber (Becton Dickinson) consists of Falcon cell culture inserts containing 8-μm pore size polyethylene terephthalate (PET) membrane coated with Matrigel basement membrane matrix. Diff Quick staining solution was from International Reagents (Kobe, Japan). Every reagent was resuspended as indicated by its manufacturer.

### Conditioned medium

In every experiment, cells were initially plated at a density of $2 \times 10^6$ per 5 ml per flask in RPMI-1640 supplemented with 5% FBS for 24 h. After this incubation, the cells were washed once with serum-free medium. The medium was then replaced with serum-free RPMI-1640 and the cells were incubated for 24 h at 37°C. If an experiment involved the addition of colony-stimulating factors, the latter were added after the wash with serum-free medium. Culture medium was collected and centrifuged at 300 ***g*** for 10 min to remove cell debris. Conditioned medium was concentrated ten fold using an Amicon ultrafiltration apparatus (MA, USA).

### Zymography

Zymographic analysis was performed as previously reported (Murphy and Crabbe, 1995). Briefly, proteinase activity was assayed by electrophoresing an aliquot of conditioned medium on 9% sodium dodecyl sulphate (SDS)-polyacrylamide gel co-polymerized with 0.1% gelatin (gelatin zymography) or 0.1% gelatin and 13 μg $ml^{-1}$ plasminogen (PA zymography). After electrophoresis at 4°C, gels were washed in 2.5% Triton X-100 for 30 min to remove SDS. Gelatin gels were then incubated for 16 h at 37°C in 50 mM Tris-HCI, pH 8.0, 0.2 M sodium chloride, 10 mM calcium chloride and Triton X-100 (activation buffer). Gels containing plasminogen were incubated in 0.1 M glycine–sodium hydroxide, pH 8.3, for 16 h at 37°C. Gels were stained with 1% Coomassie brilliant blue R-250 and proteinase activity in conditioned medium was visualized as a clear zone against a blue background.

Organomercurial activation of prometalloproteinases was achieved by incubating conditioned medium with 1 mM APMA for 1 h at 37°C before adding reducing agent-free sample buffer, and then processed for zymography. To study inhibition of metalloproteinase activity, samples were electrophoresed through gelatin-containing gels, washed twice for 30 min in 2.5% Triton X-100, rinsed with 10 mM Tris-HCI, pH 8.0, and incubated in activation buffer in the presence or absence of 1 mM 1,10-phenanthroline or 10 mM EDTA for 16 h at 37°C. The gels were then stained with Coomassie brilliant blue R-250 as described above.

Enzyme activities in the gel slabs were quantified using image analysis (image analysis program NIH Image 1.52 Macintosh), which quantified both the surface area and the intensity of lysis bands. The amount of proteinase activity is expressed as arbitrary densitometric units relative to the control cells assigned a value of 1.0.

### Western Blot

Aliquots of conditioned medium were applied to SDS-PAGE under reducing conditions. The separated proteins were transferred to a nitrocellulose membrane. The membrane was blocked overnight in 5% skimmed milk in Tris-buffered saline/Tween 20 solution. After multiple washes in Tris-buffered saline/Tween 20 solution, the membranes were incubated with the corresponding primary antibodies (1: 1000 dilution for 2 h). Multiple washes in Tris-buffered saline/Tween 20 solution were performed before application of the secondary anti-mouse antibody (1: 1000 dilution for 1 h). After further washes in Tris-buffered saline/Tween 20 solution, MMP-2 and MMP-9 protein were detected using the ECL Western blotting analysis system.

### Invasion assay

Invasiveness was measured by use of the Biocoat Invasion Chamber (Pei et al, 1996). Before seeding, cells were starved in serum-free medium for 24 h and subsequently incubated with RPMI-1640 plus 10% NuSerum in the presence of various concentrations of CSFs for another 24 h. NuSerum replaced FBS in the invasion assay because it contains standardized growth factors and

*© Cancer Research Campaign 1999* 

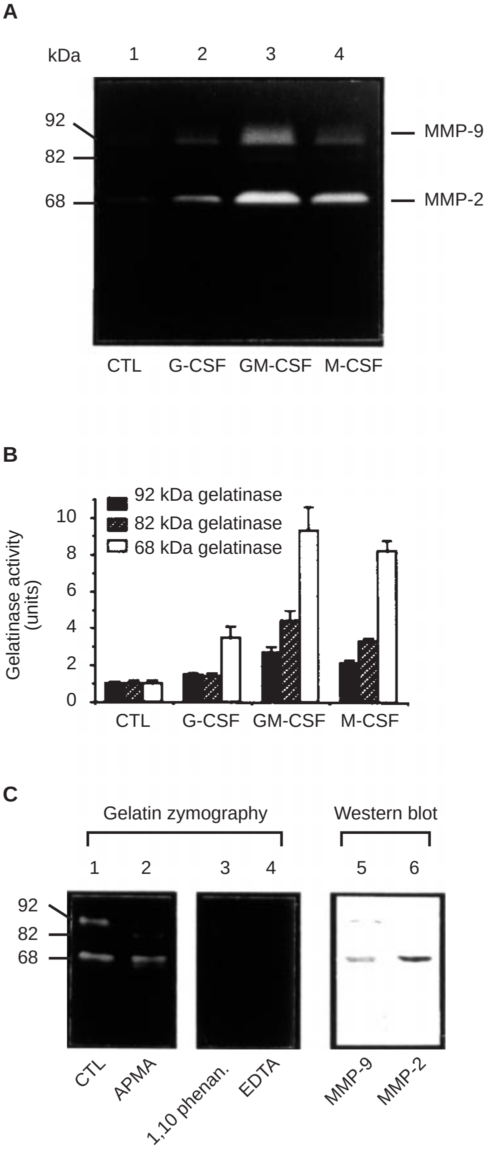

**Figure 1** (**A**) Effect of CSFs on gelatinase activity in PC-9 cells. After incubation with G-CSF (1 µg ml$^{-1}$), GM-CSF (10 ng ml$^{-1}$) or M-CSF (1000 IU ml$^{-1}$), conditioned media were collected and analysed by gelatin zymography and compared with untreated control (lane 1). (**B**) Quantitative analysis of gelatinase activity in the conditioned medium of PC-9 cells. The gelatinolytic bands of 92 kDa, 82 kDa and 68 kDa were scanned in three positions by densitometry, and the peak areas were averaged to give the values presented. The data are shown as mean values ± s.d. of three different experiments. Units of peak area are arbitrary. (**C**) Confirmation of MMP-2 and MMP-9 secreted by PC-9 cells. The conditioned media were incubated (37°C) without (lane 1) or with 1 mM APMA for 1 h (lane 2). The samples were then analysed by gelatin zymography. Zymogram of PC-9 cells was incubated in the presence of 1, 10-phenanthroline (1 mM) (lane 3) or EDTA (10 mM) (lane 4) for 16 h at 37°C. The conditioned medium was analysed by Western blotting using monoclonal antibodies to MMP-9 (lane 5) and MMP-2 (lane 6) respectively less protease inhibitors. Cells were removed from the cell culture flask and suspended in NuSerum. Two millilitres of cell suspension ($1 \times 10^5$ cells ml$^{-1}$) was placed in the upper well. Lower wells were also filled with RPMI-1640 supplemented with 10% NuSerum and the plates were incubated for 24 h. The cells that invaded the Matrigel-coated filters and floated in the medium of

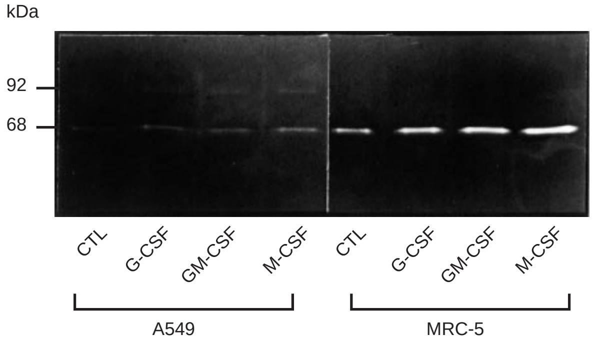

**Figure 2** Effect of CSFs on gelatinase activity in A549 and MRC-5 cells. After incubation with G-CSF (1 µg ml$^{-1}$), GM-CSF (10 ng ml$^{-1}$) or M-CSF (1000 IU ml$^{-1}$), conditioned media derived from A549 cells or MRC-5 cells were collected and analysed by gelatin zymography and compared with untreated control the lower well were collected. These samples were stored in a culture tube on ice and the lower well was filled with 2 mM EDTA. After 10 min incubation at room temperature, cells which had passed through the filter and adhered to the bottom surface were collected. After the cells were trapped with a Cell Culture Insert (3 µm), they were stained with Diff Quick and counted. The invasiveness of the cells was evaluated as per cent invasion. It was derived from the following formula:

$$\frac{\text{total no. of invading cells (lower well sample)}}{\text{total no. of seeded cells (upper well sample)}} \times 100$$

### Statistical analysis

The data were analysed for significance using Student's *t*-test.

### RESULTS

### Effect of CSFs on MMPs secretion and activity

To study the effects of CSF on MMP-2 and MMP-9 secretion, conditioned media of the tumour cells cultured in the presence or absence of different CSFs for 24 h were subjected to zymography (Figure 1A). Two bands with molecular weights of 92 000 and 68 000 were common to PC-9 cells treated with or without CSFs. The enzyme activities of $M_r$ 68 000 and $M_r$ 92 000 were significantly enhanced in the presence of CSFs. The levels of the 68-kDa and the 92-kDa gelatinase activity in the conditioned media were quantitated by densitometric analysis. Figure 1B shows a 3.5-fold increase in 68-kDa gelatinase after treatment with G-CSF, and a 9.3- and 8.2-fold increase after treatment with GM-CSF and M-CSF respectively. To a much lesser degree, a 1.5-, 2.7- and 2.1-fold increase in 92-kDa gelatinase occurred, in response to G-CSF, GM-CSF and M-CSF respectively. Further, in the presence of CSFs, an additional band at $M_r$ 82 000 was also found. This was consistent with the band caused by APMA treatment (Figure 1C). Quantitative analysis of the 82-kDa gelatinase revealed a 1.4-, 4.4- and 3.3-fold increase in response to G-CSF, GM-CSF and M-CSF. Doses of CSFs we chose were based on previous studies (Young et al, 1993; Harmenberg et al, 1994; Chambers et al, 1995; Pei et al, 1996) and the data derived from the invasion assay in the present report.

When the effects of various proteinase inhibitors on these activities were examined, 1 mM 1, 10-phenanthroline and 10 mM

© Cancer Research Campaign 1999

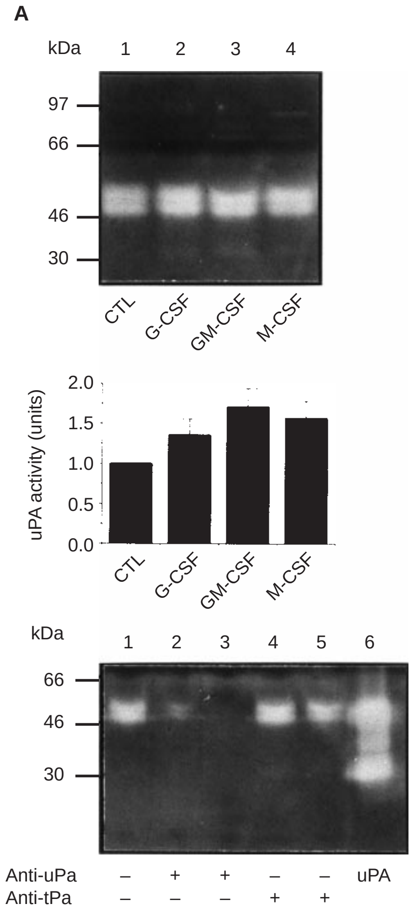

**Figure 3** (**A**) Effect of CSFs on uPA activity in PC-9 cells. Media samples from the same experiment as Figure 1A were analysed by plasminogen-activator zymography as described in Materials and methods. (**B**) Quantitative analysis of uPA activity in PC-9 cells. The plasminogen-dependent gelatinolytic bands at 48–55 kDa were scanned in three positions by densitometry, and the peak areas were averaged to give the values presented. The data are shown as mean values ± s.d. of three different experiments. Units of peak area are arbitrary. (**C**) Confirmation of uPA in the conditioned medium of PC-9 cells. The conditioned medium was incubated without (lane 1) or with polyclonal antibodies to uPA (30 μg $ml^{-1}$, lane 2; 385 μg $ml^{-1}$, lane 3) and tPA (30 μg $ml^{-1}$, lane 4; 385 μg $ml^{-1}$, lane 5) for 1 h at 37°C, and then analysed by plasminogen-activator zymography. Standard human uPA (0.1 unit) was shown in lane 6

EDTA, but not inhibitors for serine, cysteine and aspartic proteinases, inhibited completely the activities of $M_r$ 92 000, 82 000 and 68 000 bands, showing that all gelatinolytic enzymes were metalloproteinases (Figure 1C, lanes 3 and 4).

The electrophoretic mobilities of the $M_r$ 92 000 and $M_r$ 68 000 enzymes on the zymogram were identical to those of MMP-9 and MMP-2 respectively (Murphy and Crabbe, 1995). Western blot analysis of the conditioned medium was performed using anti-MMP-9 and MMP-2 monoclonal antibodies. Immunoblot labelled with anti-MMP-2 antibody is shown in lane 6 of Figure 1C. The medium contained an immunoreactivity band at 68 kDa corresponding in position to the major gelatinolytic band demonstrated by zymography. Immunoblot labelled with anti-MMP-9 antibody (lane 5) showed two bands at 92 kDa and 68 kDa, the former of which may represent MMP-9 whereas the latter is co-migrated with MMP-2 and may be the result of crossreaction of the anti-MMP-9 with the more highly expressed MMP-2.

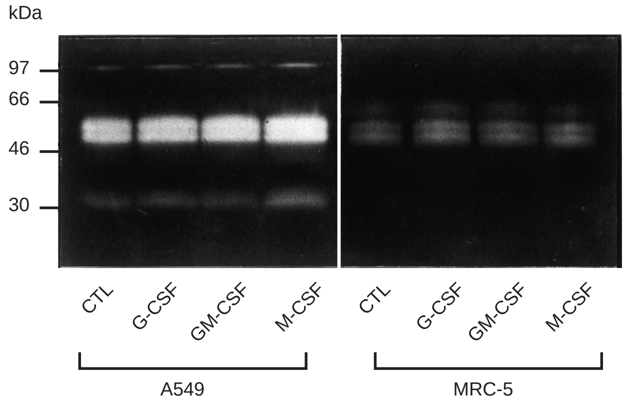

**Figure 4** Effect of CSFs on uPA activity in A549 and MRC-5 cells. Media samples from the same experiment as Figure 2 were analysed by plasminogen-activator zymography as described in Materials and methods

To further confirm the stimulatory effect of CSFs on gelatinase secretion, similar experiments were performed by using two other cell lines, one was A549, a human lung adenocarcinoma cell line, and the other was MRC-5, a human lung fibroblast cell line. The A549 cells showed a similar response of MMP-2 secretion with CSFs stimulation (Figure 2). Densitometric analysis revealed a two-, 1.7- and 4.9-fold increase in the 68-kDa gelatinase in response to G-CSF, GM-CSF and M-CSF respectively. Although the 92-kDa gelatinase activity secreted by the A549 cells was weak, the stimulatory effects of CSFs on MMP-9 can still be identified (Figure 2). Significant stimulatory effect of CSFs on MMP-2 secretion was also found in the MRC-5 cells.

### Effect of CSFs on uPA secretion and activity

Plasminogen-activator zymography was used to detect the effect of CSFs on plasminogen activator (PA) secretion and activity. Figure 3A showed that 24 h of incubation of PC-9 cells with G-CSF, M-CSF or GM-CSF in serum-free medium resulted in an increase of a 48–55 kDa plasminogen-dependent gelatinolytic activity that co-migrated with the uncleaved two chain form of human uPA. Densitometric analysis showed that 48–55-kDa PA activities were enhanced 1.4-, 1.7- and 1.6-fold in response to G-, GM- and M-CSF (Figure 3B). A parallel increase of plasminogen-dependent gelatinolytic bands at the sizes of 92 kDa and 78 kDa was also observed after CSFs treatment of PC-9 cells and may represent two types of uPA/PAI complex (Niedbala and Stein, 1991; Niedbala and Picarella, 1991). In addition, CSFs treatment also induced a faint band at a molecular weight of 33 kDa, which corresponds to a low-molecular-weight chain of the uPA. Confirmation of uPA activity was shown in Figure 3C. Polyclonal antibody (30 μg $ml^{-1}$) to uPA inhibited the PA activity by more than 80%, further, 385 μg $ml^{-1}$ anti-uPA antibody completely inhibited the PA activity of PC-9 cells. However, polyclonal antibody to tPA had no effect on the PA activity (densitometric data not shown). The gelatinolytic activity observed represented true PA activity because, like the uPA standard and all other lytic bands co-migrating with it, these bands failed to show gelatinolytic activity in replicate gels polymerized in the absence of plasminogen.

The A549 cells showed similar response of 48–55 kDa PA activities with CSFs stimulation. Densitometric analysis revealed

© Cancer Research Campaign 1999

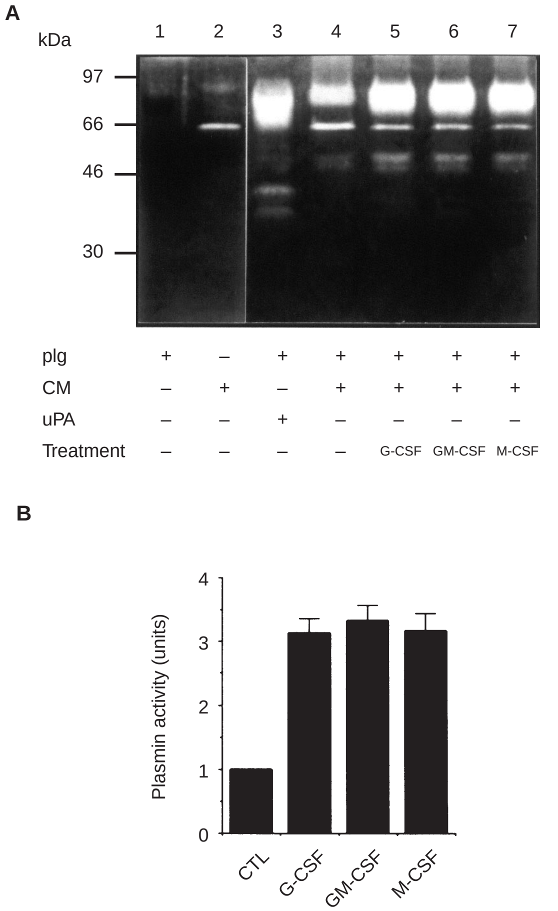

**Figure 5** (**A**) Activation of plasminogen by the conditioned medium of PC-9 cells. Media samples from the same experiment as Figure 3A incubated for 4 h with plasminogen (50 µg ml$^{-1}$) (lane 4–7) or without plasminogen (lane 2) were analysed by gelatin zymography. As controls, plasminogen was incubated in culture wells with no conditioned medium (lane 1) or incubated with human uPA (1 unit) to generate plasmin (lane 3). (**B**) Quantitative analysis of plasmin activity. The 70–90 kDa gelatinolytic bands from lanes 4–7 of Figure 5A were scanned in three positions by densitometry, and the peak areas were averaged to give the values presented. The data are shown as mean values ± s.d. of three different experiments

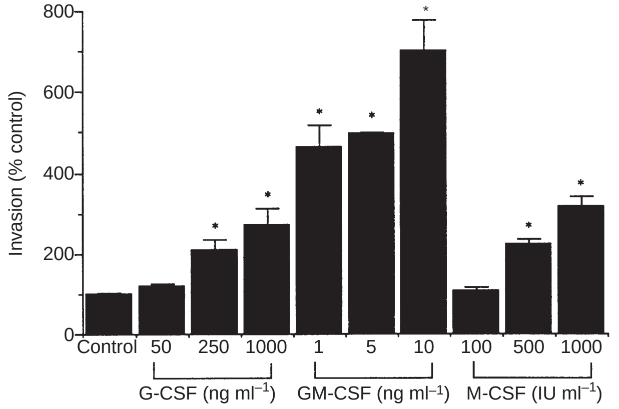

**Figure 6** Effect of CSFs on invasion by PC-9 cells. The cells were starved for 24 h, incubated with or without CSFs for 24 h and then seeded into a matrigel-precoated invasion chamber. After 24 h, invading cells were counted. The number of invading cells is expressed as the percentage of cells pretreated with the medium only (control). Values represent the mean ± s.e. ($n$ = 12). *$P$ < 0.05 compared with controls that a 1.3-, 1.4- and 1.6-fold increase in 48–55 kDa PA activity in response to G-CSF, GM-CSF and M-CSF respectively. Treatment with CSFs, especially M-CSF, also induced significant increase in 33 kDa and 92 kDa PA activities by A549 cells (Figure 4). Similar response of uPA activity with CSFs stimulation was also found in the MRC-5 cells.

### Direct plasminogen activation by the conditioned medium of PC-9 cells

The ability of PC-9 cells pretreated with CSFs to stimulate the activation of plasminogen to plasmin was determined by gelatin zymography (Figure 5A). A prominent band of lysis corresponding to plasmin (approximately 70–90 kDa) was present in the conditioned medium incubated for 4 h with plasminogen. It co-migrated with the plasmin (lane 3) prepared by incubating human plasminogen with human uPA (10:1) at 37°C for 1 h. Other minor additional bands with molecular weights of 38 000–45 000 may be due to plasmin fragments (Koshikawa et al, 1992; Roche et al, 1983). CSFs treatment stimulated significantly the conversion of plasminogen to plasmin. This was only faintly visible in the conditioned medium from PC-9 cells incubated without plasminogen (lane 2) and was absent from the control (lane 1), in which plasminogen was incubated in tissue culture wells containing neither the conditioned medium nor standard uPA. Densitometric analysis revealed that plasmin activity was increased 3.1-, 3.3- and 3.2-fold in response to G-, GM- and M-CSF treatment (Figure 5B). The conditioned medium catalysed the conversion of plasminogen to plasmin in the absence of exogenous PAs, indicating that PC-9 cells can amplify extracellular proteolytic capability by zymogen activation.

### Effect of CSFs on invasion by the cancer cells

An estimate of the invasive property of a cell type can be obtained by measuring the ability of the cells to migrate through matrigel. We measured the ability of PC-9 cells to migrate through a film of matrigel and the effects of CSFs on the migration rate. As shown in Figure 6, G-CSF, GM-CSF and M-CSF promoted invasion in a dose-dependent manner. At concentrations of 1000 ng ml$^{-1}$, 10 ng ml$^{-1}$ and 1000 IU ml$^{-1}$, G-CSF, GM-CSF and M-CSF stimulated the invasion 2.7-, 7.0- and 3.2-fold respectively. Similarly, G-CSF (1000 ng ml$^{-1}$), GM-CSF (10 ng ml$^{-1}$) and M-CSF (1000 IU ml$^{-1}$) increased the invasiveness of A549 cells 2.1-, 1.6- and 3.8-fold respectively. The invasivenesses of the PC-9 and A549 cells are 0.054% and 0.064% in the absence of CSFs. They were demonstrated to be low invasive cell lines (Pei et al, 1996).

### DISCUSSION

G-CSF, GM-CSF and M-CSF are currently being used in clinics to alleviate chemotherapy-induced bone marrow toxicity (Bukowski et al, 1994; Vose and Armitage, 1995). Although we and other researchers have demonstrated that CSFs may promote invasion by human tumour cells, the mechanisms of this effect remains unknown (Young et al, 1993; Chambers et al, 1995; Pei et al, 1996).

Previous studies have established a correlation between the invasive activity of tumour cells and the production of proteinases

 © Cancer Research Campaign 1999

capable of degrading extracellular matrix components (Stetler-Stevenson, 1990; Testa and Quigley, 1990; Mignatti and Rifkin, 1993; Stearns and Wang, 1994). In the present study, we reported that G-CSF, GM-CSF or M-CSF promoted invasion by human lung cancer cells. They stimulated the secretion and activity of serine proteinase, uPA, and metalloproteinases, MMP-9 and MMP-2, by the cancer cells as well as human lung fibroblast cells. The conditioned medium from the cancer cells treated with CSFs stimulated significantly activation of plasminogen to plasmin.

Gelatinase A (72-kDa or 68-kDa type IV collagenase/MMP-2) and gelatinase B (92 kDa or 90 kDa type IV collagenase/MMP-9), two distinct matrix metalloproteinases (MMPs) which both degrade basement membrane type IV collagen, participate in basement breakdown by migrating tumour cells. Many human tumours, including lung cancer, have been found to express the MMP-9 and MMP-2. Correlation between the secretion of these enzymes and the metastatic potential in vivo or invasiveness in vitro has been reported for many tumours. Synthesis and secretion of MMPs and their inhibitors are regulated by cytokines and some less well-defined factors (Stetler-Stevenson, 1990; Liotta et al, 1991; Mignatti and Rifkin, 1993). Our data showed that the MMP-9 and MMP-2 enzyme activities produced by either lung cancer cells or lung fibroblast cells were enhanced significantly in the presence of CSFs. Similar results were reported by Shimizu et al, (1996), in which they found secretion of gelatinases A and B by a murine colon carcinoma cell line was augmented by GM-CSF, however, no detail was reported.

Like other members of the MMP family, MMP-2 and MMP-9 are secreted as latent proenzymes and must undergo proteolytic cleavage of an amino-terminal domain, with subsequent loss of molecular weight, in order to be catalytically active (Mignatti and Rifkin, 1993; Murphy and Crabbe, 1995). Enzyme activation is an important control step in proteolysis. In the present report, when the conditioned medium was treated with 1 mM APMA for 1 h, it activated $M_r$ 92 000 pro-MMP-9 to the $M_r$ 82 000 active form. Pro-MMP-2 was also activated to the $M_r$ 62 000 forms. Of particular interest, CSFs treatment also activated part of the pro-MMP-9 produced by PC-9 cells to the $M_r$ 82 000 form – this was consistent with the active form of MMP-9 (Shapiro et al, 1995).

uPA, a major type of the plasminogen activator, is a serine protease which cleaves plasminogen to yield plasmin, an extracellular protease of broad specificity (Testa and Quigley, 1990; Mignatti and Rifkin, 1993). It is synthesized and secreted by tumour cells and normal cells and interacts with a specific cell-surface receptor (uPAR), thereby leading to focalized proteolysis. The activity of uPA is controlled by the plasminogen activator inhibitor type 1 (PAI-1) which triggers internalization of uPAR-bound uPA (Cubellis et al, 1990). The expression of PA and its inhibitors can be modulated by a number of biological agents, including tumour promoters, growth factors and cytokines. Its expression in human tumour cells was reported to correlate with their invasiveness in vitro and metastatic potential in vivo (Testa and Quigley, 1990; Mignatti and Rifkin, 1993).

In addition to the stimulation of the $M_r$ 48 000–55 000 form of uPA, CSFs also enhanced the secretion of $M_r$ 92 000, 76 000 and 33 000 plasminogen-dependent gelatinolytic bands in the cancer cells, whereas the $M_r$ 92 000 form may represent a uPA/PAI-1 complex with residual plasminogen activating activity based on immunoprecipitation and Western blot analysis (Niedbala and Picarella, 1991; Koshikawa et al, 1992). The $M_r$ 76 000 species may represent an immunoreactive proteolytic breakdown product of the $M_r$ 92 000 species because it has been shown that both the $M_r$ 55 000 and 33 000 forms of uPA can form SDS stable complexes with the $M_r$ 46 000 form of PAI-1 (Hekman and Loskutoff, 1985). The $M_r$ 33 000 lytic band represents an enzymatically active proteolytic fragment of the $M_r$ 55 000 form of the uPA, based on its co-migration and immunological crossreactivity with the uPA standard (Testa and Quigley, 1990; Mignatti and Rifkin, 1993). Although we did not check the CSFs-mediated stimulation of uPA at mRNA level in this study, previous reports demonstrated GM-CSF and M-CSF augmented uPA transcripts and activity in macrophage and carcinoma cell lines, including human lung carcinoma cell lines (Hart et al, 1990; Hamilton et al, 1991; Chambers et al, 1995; Stacey et al, 1995). We also found G-CSF increased uPA transcripts in lung cancer cells (Pei et al, 1998).

Filderman et al (1992) previously reported no specific effect on invasiveness in A549 cells by GM-CSF. In the present study, we found GM-CSF also stimulated the invasion and secretion of uPA and MMP-9 by A549 cells, although the response was not as great as the PC-9 cells. These inconsistencies may result from the different invasion system used. Nevertheless, the present study reported that M-CSF induced more protease activity and more invasion than the other two factors did by A549 cells. This was in line with their results on the invasion by the A549 cells treated with M-CSF. Furthermore, we checked the effect of GM-CSF on the invasion by other lung cancer cell lines. GM-CSF was found to promote the invasion by human lung squamous cell carcinoma cell lines, LK-2 and LC-1, in vitro (Tsuruta et al, 1998). It was also reported in other cancer cell lines that GM-CSF stimulated the protease secretion and metastatic potential as well (Young et al, 1993; Shimizu et al, 1996).

The expression of uPA and MMPs is mainly modulated at the transcriptional level by oncogenes, tumour promoters and cytokines (Michel and Quertermous, 1989; Mignatti and Rifkin, 1993; Ries and Petrides, 1995; Stacey et al, 1995). Because of the discrepant structures of these enzymes and their inhibitors, especially striking differences of their promoters, regulation of these proteins is quite discordant. It is possible that CSFs modulate their expression to different extents. Nevertheless, a similar trend in the stimulation of uPA, MMPs and invasion (GM-CSF> M-CSF>G-CSF) was found in the PC-9 cells. Because the PA/plasmin system plays a central role in ECM degradation, even the relatively small amount of uPA stimulated by CSFs may exert a significant effect on invasion in vivo.

Concerning the stimulatory effect of CSFs on uPA and MMPs secretion by lung fibroblast cell line MRC-5, our results are consistent with the previous reports in which uPA expression was enhanced by interleukin 1, another one of the haematopoietic growth factors (Michel and Quertermous, 1989). This may suggest a role of these enzymes and this cell type in the response to inflammation.

Because CSFs stimulated the production of uPA by the cancer cells, the ability of the conditioned medium to activate plasminogen in the absence of exogenous PAs was determined. The conditioned medium from PC-9 cells treated with CSFs stimulated the conversion of plasminogen to plasmin, providing a direct demonstration of the ability of enhanced PC-9 uPA to increase plasmin-dependent proteolysis.

In summary, we showed that the capacity of the lung cancer cells treated with CSFs to invade through reconstituted basement membrane (Matrigel) was increased significantly. The enhanced

© Cancer Research Campaign 1999

invasive behaviour of the cancer cells stimulated by CSFs was well correlated with the increase in MMPs and uPA activities. Tumour cell-associated uPA efficiently activated plasminogen in solution, resulting in the formation of soluble plasmin. These data suggest that the enhanced production of extracellular matrix-degrading proteinases by the cancer cells in response to CSFs treatment may represent a biochemical mechanism which promotes the invasive behaviour of the cancer cells.

### ACKNOWLEDGEMENT

This work was supported by the Sasakawa Health Science Foundation.

### REFERENCES

Bukowski RM, Budd GT, Gibbons JA, Bauer RJ, Childs A, Antal J, Finke J, Tuason L, Lorenzi V, McLain D et al (1994) Phase I trial of subcutaneous recombinant macrophage colony-stimulating factor: clinical and immunomodulatory effects. *J Clin Oncol* **12**: 97–106

Chambers SK, Wang Y, Gertz RE and Kacinski BM (1995) Macrophage colony-stimulating factor mediates invasion of ovarian cancer cells through urokinase. *Cancer Res* **55**: 1578–1585

Cubellis MV, Wun TC and Blasi F (1990) Receptor-mediated internalization and degradation of urokinase is caused by its specific inhibitor PAI-1. *EMBO J* **9**: 1079–1085

Filderman AE, Bruckner A, Kacinski BM, Deng N and Remold HG (1992) Macrophage colony-stimulating factor (CSF-1) enhances invasiveness in CSF-1 receptor-positive carcinoma cell lines. *Cancer Res* **52**: 3661–3666

Hamilton JA, Vairo G, Knight KR and Cocks BG (1991) Activation and proliferation signals in murine macrophages. Biochemical signals controlling the regulation of macrophage urokinase-type plasminogen activator activity by colony-stimulating factors and other agents. *Blood* **77**: 616–627

Harmenberg J, Hoglund M and Hellstrom-Lindberg E (1994) G- and GM-CSF in oncology and oncological haematology. *Eur J Haematol* **52**: 1–28

Hart PH, Vitti GF, Burgess DR, Whitty GA, Royston K and Hamilton JA (1990) Activation of human monocytes by granulocyte-macrophage colony-stimulating factor: increased urokinase-type plasminogen activator activity. *Blood* **77**: 841–848

Hekman CM and Loskutoff DJ (1985) Endothelial cells produce a latent inhibitor of plasminogen activators that can be activated by denaturants. *J Biol Chem* **260**: 11581–11587

Herlyn M and Malkowicz SB (1991) Regulatory pathways in tumor growth and invasion. *Lab Invest* **65**: 262–27

Jeffers M, Rong S and Vande Woude GF (1996) Enhanced tumorigenicity and invasion-metastasis by hepatocyte growth factor/scatter factor-met signalling in human cells concomitant with induction of the urokinase proteolysis network. *Mol Cell Biol* **16**: 1115–1125

Koshikawa N, Yasumitsu H, Umeda M and Miyazaki K (1992) Multiple secretion of matrix serine proteinases by human gastric carcinoma cell lines. *Cancer Res* **52**: 5046–5053

Liotta LA, Steeg PS and Stetler-Stevenson WG (1991) Cancer metastasis and angiogenesis: an imbalance of positive and negative regulation. *Cell* **64**: 327–336

Michel JB and Quertermous T (1989) Modulation of mRNA levels for urinary- and tissue-type plasminogen activator and plasminogen activator inhibitors 1 and 2 in human fibroblasts by interleukin 1. *J Immunol* **143**: 890–895

Mignatti P and Rifkin DB (1993) Biology and biochemistry of proteinases in tumor invasion. *Physiol Rev* **73**: 161–195

Murphy G and Crabbe T (1995) Gelatinases A and B. *Methods Enzymol* **248**: 471–528

Niedbala MJ and Picarella MS (1991) Tumor necrosis factor induction of endothelial cell urokinase-type plasminogen activator mediated proteolysis of extracellular matrix and its antagonism by γ-interferon. *Blood* **79**: 678–687

Niedbala MJ and Stein M (1991) Tumor necrosis factor induction of urokinase-type plasminogen activator in human endothelial cells. *Biomed Biochim Acta* **50**: 427–436

Pei XH, Nakanishi Y, Takayama K, Yatsunami J, Bai F, Kawasaki M, Wakamatsu K, Tsuruta N, Mizuno K and Hara N (1996) Granulocyte-colony stimulating factor promotes invasion by human lung cancer cell lines in vitro. *Clin Exp Metastasis* **14**: 351–357

Pei XH, Nakanishi Y, Takayama K, Bai F, Kawasaki M, and Hara N (1998) G-CSF increases secretion of urokinase-type plasminogen activator by human lung cancer cells. *Clin Exp Metastasis* (in press)

Petersen LC, Lund LR, Nielsen LS, Dano K and Skriver L (1988) One chain urokinase-type plasminogen activator from human sarcoma cells is a proenzyme with little or no intrinsic activity. *J Biol Chem* **263**: 11189–11195

Ries C and Petrides PE (1995) Cytokine regulation of matrix metalloproteinase activity and its regulatory dysfunction in disease. *Biol Chem Hoppe-Seyler* **376**: 345–355

Roche PC, Campeau JD and Shaw ST (1983) Comparative electrophoretic analysis of human and porcine plasminogen activators in SDS-polyacrylamide gels containing plasminogen and casein. *Biochim Biophys Acta* **745**: 82–89

Shapiro SD, Fliszar CJ, Broekelmann TJ, Mecham RP, Senior RM and Welgus HG (1995) Activation of the 92-kDa gelatinase by stromelysin and 4-aminophenylmercuric acetate. *J Biol Chem* **270**: 6351–6356

Shimizu S, Nishikawa Y, Kuroda K, Takagi S, Kozaki K, Hyuga S, Saga S and Matsuyama M (1996) Involvement of transforming growth factor β1 in autocrine enhancement of gelatinase B secretion by murine metastatic colon carcinoma cells. *Cancer Res* **56**: 3366–3370

Stacey KJ, Fowles LF, Colman MC, Ostrowski MC and Hume DA (1995) Regulation of urokinase-type plasminogen activator gene transcription by macrophage colony-stimulating factor. *Mol Cell Biol* **15**: 3430–3441

Stearns ME and Wang M (1994) Immunoassays of the metalloproteinase (MMP-2) and tissue inhibitor of metalloproteinase (TIMP 1 and 2) levels in noninvasive and metastatic PC-3 clones: effects of taxol. *Oncol Res* **6**: 195–201

Stetler-Stevenson WG (1990) Type IV collagenases in tumor invasion and metastasis. *Cancer Metastasis Rev* **9**: 289–303

Testa JE and Quigley JP (1990) The role of urokinase-type plasminogen activator in aggressive tumor cell behavior. *Cancer Metastasis Rev* **9**: 353–367

Teti A, De Giorgi A, Spinella MT, Migliaccio S, Canipari R, Onetti Muda A and Faraggiana T (1997) Transforming growth factor-beta enhances adhesion of melanoma cells to the endothelium in vitro. *Int J Cancer* **72**: 1013–1020

Tsuruta N, Yatsunami J, Takayama K, Nakanishi Y, Ichinose Y and Hara N (1998) Granulocyte–macrophage colony-stimulating factor stimulates tumor invasiveness in squamous cell lung cancer. *Cancer* (in press)

Vose JM and Armitage JO (1995) Clinical applications of hematopoietic growth factors. *J Clin Oncol* **13**: 1023–1035

Weimar IS, De Jong D, Muller EJ, Nakamura T, Van Gorp J, De Gast GC and Gerritsen WR (1997) Hepatocyte growth factor/scatter factor promotes adhesion of lymphoma cells to extracellular matrix molecules via alpha 4 beta 1 and alpha 5 beta 1 integrins. *Blood* **89**: 990–1000

Young MRI, Lozano Y, Djordjevic A, Devata S, Matthews J, Young ME and Wright MA (1993) Granulocyte–macrophage colony-stimulating factor stimulates the metastatic properties of Lewis lung carcinoma cells through a protein kinase A signal-transduction pathway. *Int J Cancer* **53**: 667–671

Zhang M, Wang MH, Singh RK, Wells A and Siegal GP (1997) Epidermal growth factor induces CD44 gene expression through a novel regulatory element in mouse fibroblasts. *J Biol Chem* **272**: 14139–14146

© Cancer Research Campaign 1999
